# A survey of parental health-seeking behaviour, knowledge, and expectations around ear infection symptoms in children

**DOI:** 10.3399/BJGPO.2025.0131

**Published:** 2026-03-11

**Authors:** Catherine V Hayes, Haroon Ahmed, Julie V Robotham, Neville Q Verlander, Donna M Lecky

**Affiliations:** 1 Primary Care and Interventions Unit, AMR and HCAI division, UK Health Security Agency, Gloucester, UK; 2 Division of Population Medicine, Cardiff University, Cardiff, UK; 3 AMR Modelling and Evaluations, AMR and HCAI Division, UK Health Security Agency, Colindale, UK; 4 Statistics, Modelling and Economics Department, UK Health Security Agency, Colindale, UK

**Keywords:** child health, infectious illness, patient perspectives, otitis, primary health care

## Abstract

**Background:**

An estimated half a million UK primary care consultations are due to middle ear infections annually. In children, ear infections are one of the most common reasons for antibiotic use.

**Aim:**

To describe parents’ experiences and actions for their child’s ear infection symptoms.

**Design & setting:**

Online survey with parents of children aged ≤10 years who had suspected ear infection symptoms in the previous 12 months in England.

**Method:**

Data were collected retrospectively and through self-report. Multivariable logistic regression explored association of variables with consulting and reported prescription of antibiotics.

**Results:**

In total, 503 parents participated. Most parents perceived their child’s symptoms as mild (25.8%) or moderate (64.6%). Consulting health care was the most reported action (90.3%); 74.0% consulted within 1–2 days. Perceived severity and duration of symptoms were associated with consulting. Parents were concerned about serious illness and the need for treatment. Other factors associated with consulting were missing work (adjusted odds ratio [aOR] 4.8, 95% confidence interval [CI] = 1.6 to 14.8), childcare impacts (aOR 3.1, 95% CI = 1.0 to 9.5), and fluid in child’s ear (aOR 3.9, 95% CI = 1.48 to 10.5). Of consultors, 55.7% (253/454) reported receiving an antibiotic. Of all parents (503), 36.4% believed children always require antibiotics for ear infections.

**Conclusion:**

Most parents consult for their child’s ear infection symptoms and are prompted by impacts on daily life and perceptions of risk and treatment needs. There are knowledge gaps that, if addressed, may help to reduce primary care consultations and antibiotic use by supporting parents to manage self-limiting ear infections in children.

## How this fits in

Ear infections, while often self-limiting, can have a significant impact on children and their families and can be a common reason for consulting and being prescribed antibiotics. We found that most parents we surveyed consulted health care within 1–2 days of symptom onset and had concerns around the seriousness of the infection and need for treatment. There were some misconceptions around the effectiveness of antibiotics, and over a third of parents incorrectly believed that children always require antibiotics to recover from an ear infection. Use of evidence-based educational and shared decision-making resources in the primary care setting may help to support parents. A public education campaign aimed at parents could help empower management of self-limiting ear infections and potentially reduce use of antibiotics in children.

## Introduction

Acute otitis media (AOM) is the most frequent type of ear infection experienced in childhood.^1^ It is most prevalent in children aged <10 years^2^ and is one of the most common reasons for antibiotics being prescribed to children in primary care.^3^ Around 80% of AOM is self-limiting within 2–3 days,^4^ but antibiotics are prescribed for 85%–92% of cases that present to primary care,^5,6^ despite estimates that only 17%–19% of cases require them.^6,7^

Antibiotic use in children can cause side effects, impact the microbiome, and potentially lead to long-term adverse health outcomes.^8^ Antibiotic use is associated with antimicrobial resistance (AMR) and recent evidence suggests AMR is increasing for organisms isolated from ear infections.^9^ It is important to identify opportunities to reduce unnecessary antibiotic use in children where clinical benefit is unlikely.

There is limited evidence regarding the health-seeking behaviours of parents of children experiencing ear infections in England. Findings from a 2020 English public survey suggested that a high proportion (68%) of the population believe that antibiotics are required to treat ear infections,^10^ and this belief has persisted over time.^11^ Research from North and Central America identified high consultation rates for children with ear infections and identified perceived severity of symptoms, previous infection, and fever as factors associated with consulting.^12,13^

This study aimed to describe the knowledge, expectations, and behaviour of parents of children with symptoms of ear infection in England, including the proportion that consulted health care. The analysis aimed to identify demographic and clinical factors associated with health-seeking behaviours.

## Method

### Study design

An online cross-sectional survey collected retrospective self-reported data on parental experiences caring for a child who had symptoms of suspected ear infection (most recent case if multiple children or episodes). Survey questions (supplementary file 1) were informed by National Institute for Health and Care Excellence (NICE) guidelines and published work^10,14,15^ with input from academic and clinically active GPs and behavioural scientists. The survey questions were reviewed informally with parents of children known to the researchers who helped clarify the language and reviewed options for symptoms and health-seeking actions.

### Participants and recruitment

An independent market research agency, Basis Research, facilitated recruitment via online research panels; individuals opted to join these and were profiled during registration. The survey was displayed on the dashboard of parents and caregivers (aged ≥18 years) of children (aged ≤10 years), from England. The survey was part of a larger project exploring actions for ear infection; this appeared on 24 383 dashboards and 7886 individuals clicked on the link. Prospective participants were eligible if their child experienced ear infections symptoms in the previous 12 months. A sample of 500 participants was within resource constraints and quotas aimed for representativeness across region, age, and gender. Responders received incentives (as points) from the agency, proportional to the length of the survey. Quality checks were completed to ensure authentic responses.

### Data collection

Data collection ran from 23 November 2023 to 30 November 2023. Participants were provided with an information sheet and gave consent online, before starting the survey. The social grade of responders was classified using the National Readership Survey system.^16^ Questions were single and multiple choice, with open-ended ‘other’ options.

### Data analysis

Statistical analysis (completed in Stata version 18) included multivariable logistic regression (significance level 0.05) with likelihood ratio test. The primary outcome was whether participants consulted health care. Variables included infection presentation (symptoms, duration, severity), impact on daily life, demographics of the parent and child, and mode of consultation (for antibiotic outcome). There were very few missing data (16 responses), in only three variables. To address these, a complete-case approach to analysis was followed. To maximise observations in the statistical model, a backwards stepwise procedure was followed where variables with missing data were removed from the model one at a time, in order of amount of missing data. Variables were excluded if they were not significantly associated with the outcome; a higher significance level of 0.2 was used to ensure that a potentially significant variable was not excluded. Variables were only excluded if not substantially confounding other variables in the model (if it changed the odds ratio of one or more parameters by at least 20%). Two variables were excluded that fit these criteria (responder income and working status) and 500 observations were included in the model. The conclusions did not change when compared with the original model. The secondary outcome (antibiotic prescribed) applied to those participants who consulted a healthcare professional (*n* = 454) and the same step-wise process was completed. One variable (sleeping issues) was omitted from the statistical model of the secondary outcome owing to implausible association. An additional exploratory analysis for those who consulted a healthcare professional (*n* = 454) on what they expected from the consultation and what happened took place; a two-sample test of proportions was conducted for each of the 10 options (significance level 0.05).

### Ethics

Data were collected by an independent agency bound by the rules of the Market Research Society. Information and consent forms were developed by the agency and the authors and checked by the UK Health Security Agency (UKHSA) Research Support and Governance Office. The study received institutional ethics approval (R&D 544). Anonymous aggregated data were provided to the authors and data processing and storage complied with the UK General Data Protection Regulation and UK Data Protection Act 2018.

## Results

In total, 503 parents participated (see Supplementary Table S1 for characteristics). Most parents perceived their child’s symptoms as mild (25.8%) or moderate (64.6%). Table 1 summarises the characteristics of children, as reported by parents. Most children had experienced one (39.8%, 200/503) or two (41.9%, 211/503) symptom episodes in the previous 12 months. For their most recent infection, the most reported symptoms included fever (77.1%, 388/503) and earache (73.6%, 370/503). Most parents perceived their child’s symptoms as mild (25.8%, 130/503) or moderate (64.6%, 325/503). Specific impacts included issues sleeping (65.6%, 330/503), missing school or nursery (62.0%, 312/503), or parents taking time off work (41.6%, 209/503).

**Table 1.** Characteristics of children with ear infection symptoms reported by parents (*n* = 503)

Variable	Categories	Number	Percentage (%)
**Child age, years**	0–1	94	18.7
	2–3	160	31.8
	4–5	91	18.1
	6–10	158	31.4
**Child sex**	Male	237	47.1
	Female	266	52.9
**Number of episodes of suspected ear infection symptoms in previous 12 months**	1	200	39.8
	2	211	41.9
	3	62	12.3
	4	22	4.4
	≥5	8	1.6
**Symptoms reported (most recent episode**)	Fever	388	77.1
	Earache	370	73.6
	Redness in ear	298	59.2
	Lack of energy	298	59.2
	Pressure in ear	225	44.7
	Fluid in ear	222	44.1
	Sickness	164	32.6
	Tinnitus	106	21.1
	Dizziness	101	20.1
	Hearing loss	90	17.9
	Scaly skin around ear	83	16.5
	Balance issues	81	16.1
	Blood in ear	37	7.4
**Parents’ perception of severity of child’s symptoms**	Mild	130	25.8
	Moderate	325	64.6
	Severe	48	9.5
**Parents’ perception of duration of child’s symptoms**	<1 day	2	0.4
	1–2 days	38	7.5
	3–4 days	200	39.8
	5 –7 days	185	36.8
	8–10 days	54	10.7
	>10 days	24	4.8
**Parents’ perception of impact on their child’s daily life**	Did not interfere	47	9.3
	Prevented some of their usual activities	289	57.5
	Prevented most of their usual activities	136	27.0
	Prevented all of their usual activities	31	6.2

Symptoms reported are multiple choice where ‘yes’ selected. Parents’ perceptions of the severity, duration, and impact of their child’s symptoms relate to the most recent case.

### Actions taken for child’s suspected ear infection

**Figure 1.**
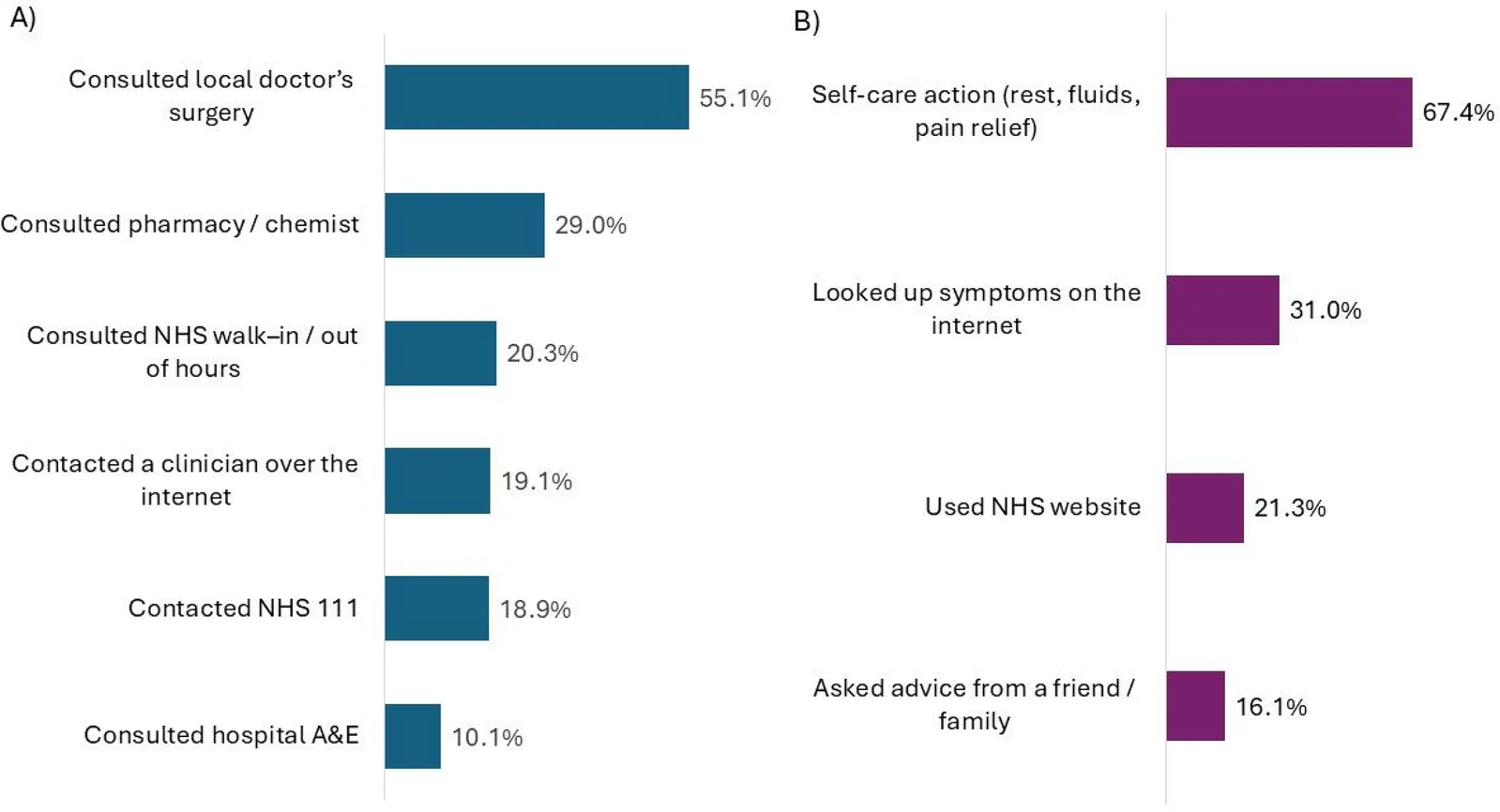
Actions taken by parent for their child with suspected ear infection symptoms (*n* = 503), including (A) consulting and (B) self-care behaviours. Consulting and self-care options were multiple choice and parents could choose more than one option. ‘Self-care action’ relates to the proportion of parents who reported at least one action of rest, increased fluid intake, and/or pain relief

Of parents, 90.3% (454/503) consulted a healthcare professional, most commonly general practice (55.1%, 277/503), followed by community pharmacy (29.0%, 146/503) (Figure 1). Of consultors (454/503), the proportion who consulted general practice was 61.0% and community pharmacy, 32.1%. Most parents (96.7%, 439/454) consulted within a week or less; 74.0% (336/454) consulted within 2 days of symptoms starting. Most consultations took place in person (61.0%, 277/454), with 16.5% (75/454) over the phone, 13.2% (60/454) a mix of in-person and remote, and 9.3% (42/454) other remote methods. At least one self-care action was reported by 67.4% (339/503) of parents, including extra rest (41.7%, 210/503), drinking more fluids (37.6%, 189/503), and taking pain relief (36.4%, 183/503).

After adjusting for variables related to clinical presentation, impact on daily life, and demographics, several factors were significantly associated with increased odds of consulting (Supplementary Table S2). Where parents missed work there were 4.8 higher odds of consulting (95% confidence interval [CI] = 1.6 to 14.8; *P* = 0.01), 3.10 higher odds if their childcare routine was impacted (95% CI = 1.0 to 9.5; *P* = 0.03), and 3.94 higher odds if there was fluid in their child’s ear (95% CI = 1.48 to 10.5, *P* = 0.003). Perceived moderate symptoms had twice the odds of consulting compared with mild symptoms (adjusted odds ratio [aOR] 2.03, 95% CI = 0.84 to 4.94); those with perceived duration of symptoms from 5–7 and ≥8 days had 8.5 (95% CI = 2.33 to 31.44) and 4.4 (95% CI = 0.79 to 24.25) higher odds of consulting compared with the shortest duration, respectively.

Of the 90.3% (454/503) who consulted, 67.0% (304/454) reported their child was prescribed treatment. Of those reporting any antibiotic (55.7%, 253/454), 68.4% (173/253) reported immediate antibiotic tablets or liquid, 17.8% (45/253) antibiotic cream or drops, and 13.8% (35/253) a delayed prescription. Pain relief and non-antibiotic ear drops were reported by 11.2% (51/454). After adjusting for variables related to clinical presentation, impact on daily life, mode of consultation, and demographics, children who experienced hearing loss had 2.24 higher odds (95% CI = 1.24 to 4.25, *P* = 0.01) of parents reporting antibiotics and the odds increased by 1.97 (95% CI = 1.09 to 3.56, *P* = 0.02) if children had fever. Consultation over the phone was associated with fewer reported antibiotics than face to face (aOR 0.27, 95% CI = 0.15 to 0.51, *P*<0.001).

### Concerns and expectations of parents

**Figure 2.**
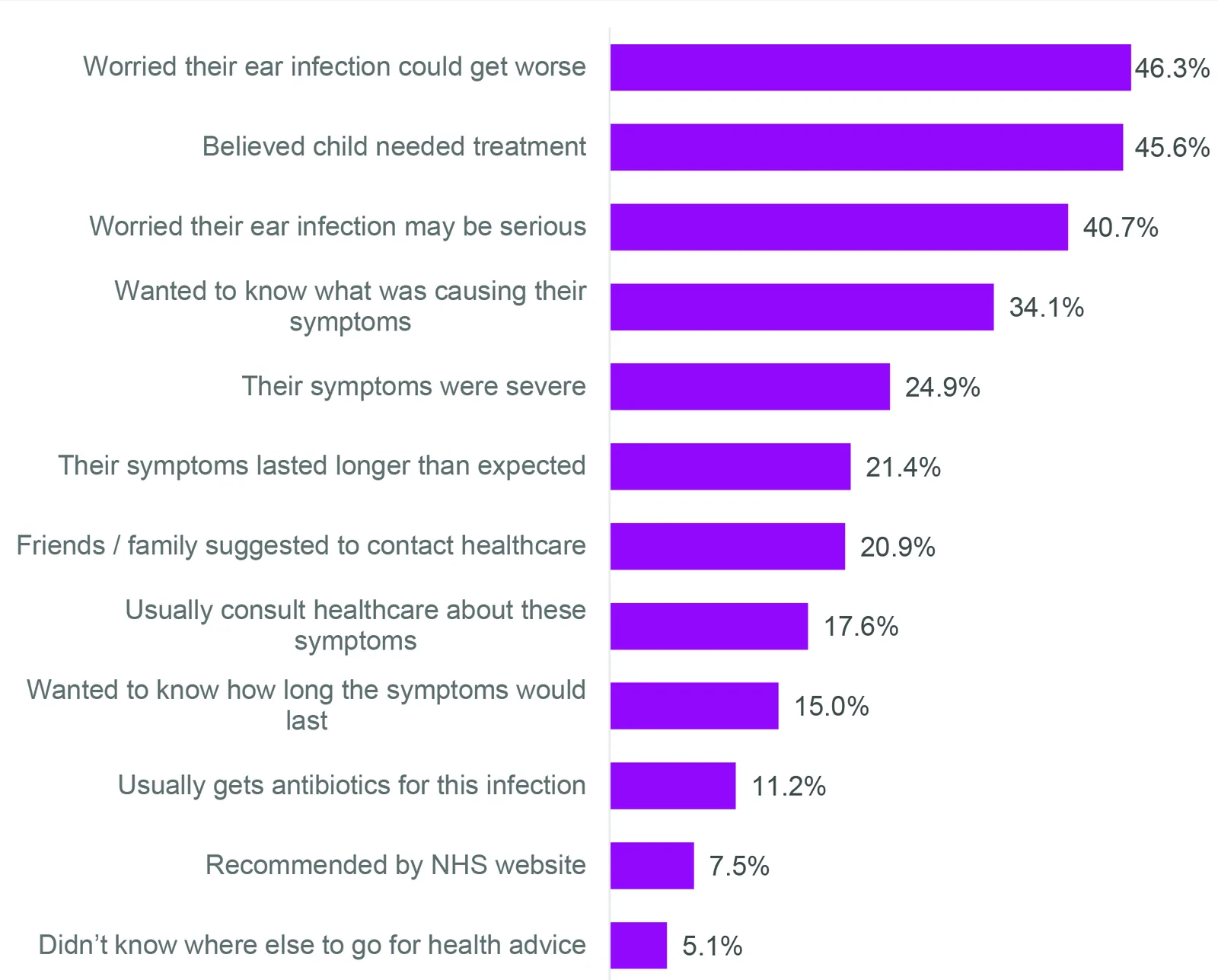
Responses from parents (*n* = 454) on their reason for consulting a healthcare professional about their child’s ear infection symptoms

Parents’ reasons for consulting (Figure 2) included concerns the infection could get worse (46.3%, 210/454), needed treatment (45.6%, 207/454), and may be serious (40.7%, 185/454). For those who did not consult (49/503), the top two reasons were belief that they could manage the child’s symptoms (63.3%, 31/49) and that symptoms were not severe enough (40.8%, 20/49).

**Figure 3.**
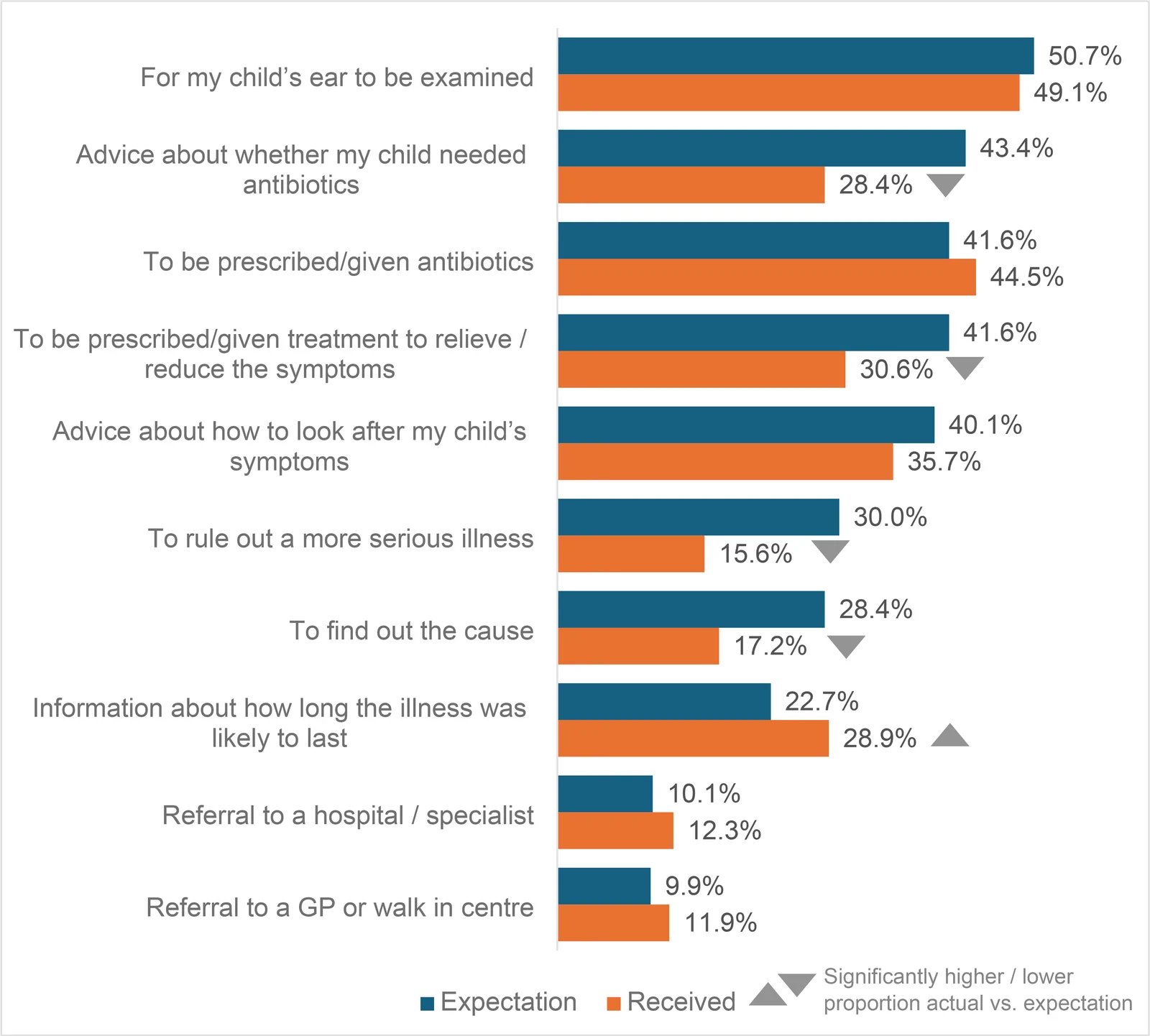
Parents’ expectations for the healthcare consultation for their child’s ear infection and what actually happened. Responses from all parents who reported consulting a healthcare professional or service for their child (*n* = 454). Analysis looked at differences in the overall proportion that expected the action or treatment and that received the action or treatment

When asked about their consultation expectations and outcomes (Figure 3), significantly more parents expected advice on whether their child needed antibiotics compared with the proportion who received advice (43.4% expected, 28.4% received, *P*<0.001). Overall, a slightly higher proportion received antibiotics than the proportion who said they expected them (44.5% versus 41.6%); however, of those who expected antibiotic treatment (189/454), 63.0% (119/189) received them. There was a significant difference between the proportion who said they expected symptomatic treatment and the proportion who said they received it (41.6% versus 30.6%, *P*<0.001).

### Parents’ beliefs about treatment and prevention of ear infections

**Figure 4.**
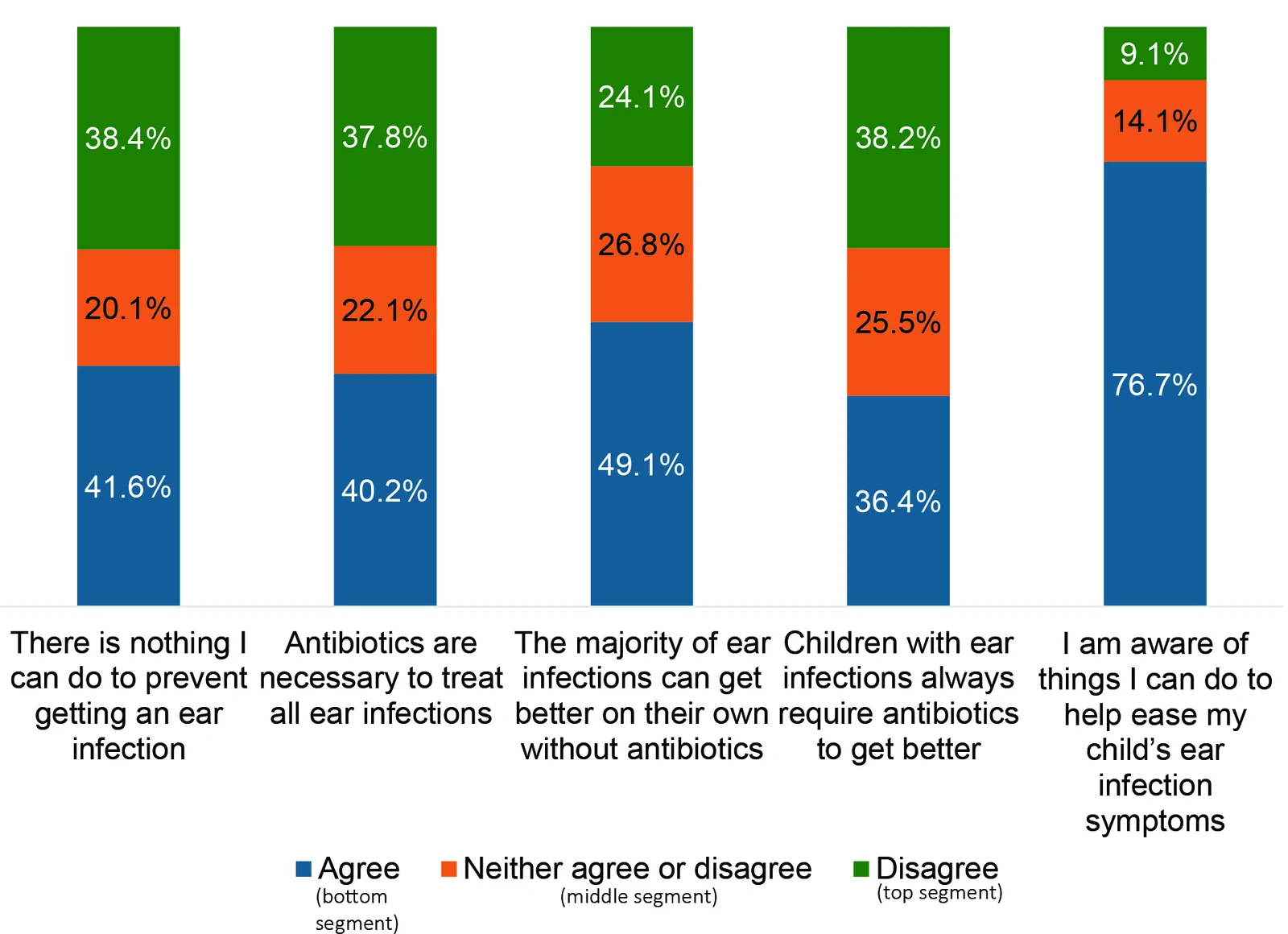
Parents’ perceived knowledge about the self-limiting nature of ear infections, effectiveness of antibiotics, and self-care behaviours

Parents had the highest agreement (76.7%, 386/503 agree) for awareness of things to help ease their child’s symptoms (Figure 4). Almost one-quarter of parents selected neither agree nor disagree across the statements on parents’ perceived knowledge about the self-limiting nature of ear infections and effectiveness of antibiotics. For the statement that most ear infections can get better without antibiotics, 49.1% (247/503) agreed, 24.1% (121/503) disagreed, and 26.8% (135/503) selected neither agree nor disagree. Over a third (36.4%, 183/503) believed children with ear infection always require antibiotics to get better.

## Discussion

### Summary

This survey provided insights into how parents manage children’s suspected ear infection symptoms in the community. Most parents consulted health care, and almost three-quarters made first contact within 1–2 days of symptoms starting. More than half of parents who consulted indicated their child received antibiotics. Consulting behaviour was associated with presentation of symptoms and impacts on daily life, and parents tended to believe that treatment was needed. While some expected antibiotics to be prescribed to their child, there may be opportunities to potentially reduce prescribing as parents’ expectations around advice and symptomatic treatment were not always met.

### Strengths and limitations

The use of a market research agency to recruit participants, rather than healthcare settings, reduced the likelihood of overestimating consulting behaviour. The study included more than 500 parents, and while quota sampling helped to reach parents across regions of England, this method could limit generalisability. Those who sign up to research panels may differ in socioeconomic status and health literacy from the wider population, and our sample had more participants from higher social grades and White ethnicity. The methodology excluded those without internet access; however, it is estimated that only 5% of the adult UK population did not have internet access in 2024.^17^

Parents self-reported ear infection and were asked about the child’s symptoms rather than type of ear infection, which helped to reach those who did not get a diagnosis. As this wasn’t clinically confirmed, there is a chance some symptoms may not have been infection. Parents with more experience of ear infection or severe cases may have been more likely to respond as these are easier to recall. This is supported by the data showing around 60% had multiple infections within the year, and over half reported symptoms lasting ≥5 days. Data on severity and duration of symptoms should be carefully interpreted as these were perceived by the parent for their child and recall may have been influenced by their actions.

The presence of missing data, albeit minor (16 responses), could mean the data may be missing other than completely at random. While a multiple imputation method would have been more robust, a stepwise approach was used owing to the number being very low, and few steps were used. Some aORs should be interpreted with caution where there are wider CIs owing to the lower number of observations in the associated categories.

Although out of scope of this study, further analysis could look at differences in prescribing for ear infections across different health settings. While we discussed survey questions informally with parents, more public involvement across the whole study design could have been impactful.

### Comparison with existing literature

A similar proportion of our sample consulted health care when compared with findings from Canada (94%) and Panama (86%).^13,18^ The impact of childhood ear infections on parents and families is well understood,^19–22^ with disruption to childcare and working days.^23,24^ Parent missed workdays had the strongest statistical relationship to the outcome in our survey. The timing of data collection occurring post-COVID-19 and during the UK ‘cost of living crisis’ may have influenced this outcome, and more research is needed on the impact of financial and job insecurity on parents' consulting behaviour. Previous research indicated patients from lower socioeconomic backgrounds are more likely to consult for respiratory tract infections (RTIs);^25^ however, we found no differences in consulting by demographics. We identified strong concerns around ear infection symptoms, in line with previous research;^26–28^ however, parents’ risk perceptions may have been influenced by the UK scarlet fever outbreak in 2023–2024.^29^

NICE recommends delayed or immediate antibiotics may be considered when children have discharge or infection in both ears (if aged <2 years).^14^ Of consulting parents in our sample, 55.7% reported their child received antibiotics. This is similar to a study investigating UK children with ear discharge from 2005 to 2019 where 57.1% were prescribed antibiotics.^30^ Our analysis found that fever was associated with greater odds of parents reporting an antibiotic for their child, similar to findings from Denmark.^31^ As others report,^32^ telephone consultations were associated with fewer reported antibiotics than face to face, which is unsurprising as clinicians would likely examine the ear in line with guidance.^14^

Deciding to prescribe antibiotics can be complex and parental knowledge and expectations is one identified area that can influence their use for children with RTIs.^33,34^ Many parents expect antibiotics for AOM.^28,35^ We found that 36.4% of parents believed that children always require antibiotics for ear infections. Overestimating the benefit of antibiotics is also common in representative UK public surveys from 2020 to 2024, with 90% believing that antibiotics work for the majority of ear infections.^11^ The authors noted declines in antibiotic knowledge post-COVID-19 pandemic, as well as increased expectations for antibiotics, symptomatic treatment, and advice about antibiotics for RTIs.^36^ They also observed an increased proportion answering ‘*don’t know*’ to questions.^11^ This was also identified in our survey, suggesting more uncertainty around antibiotic use, which may in turn lead to misunderstandings in patient and clinician interactions. There may be cases where clinicians overestimate parents’ expectations when they exhibit concerns; a survey in Germany found antibiotics were prescribed more often (70.2%) than requested (26.9%) for ear infections.^35^

### Implications for research and practice

This survey was prompted by evidence suggesting the public have high expectations for antibiotics for ear infections.^11^ Ear infections are most common in children, and paediatric antibiotic prescribing has increased since the COVID-19 pandemic, with 5.3 million prescriptions for children aged 0–14 years in 2022.^37^ We explored parental expectations and gained insights into consulting behaviour. As parents are more uncertain about antibiotic use following the pandemic, prescribers could explain their decision making, so patients understand why antibiotics were or were not prescribed. Parents require confidence in monitoring symptoms (watchful waiting) and identifying serious symptoms to know when to consult next time; national campaigns focused on parents could be beneficial. We found differences in parental expectation and provision of symptomatic treatment. Since 2022, NICE has recommended anaesthetic and analgesic ear drops as an alternative to antibiotics for children without eardrum perforation. Further work planned by the authors includes describing use of antibiotics and licensed eardrops through electronic health records. Further research could examine parents’ self-management strategies and their effectiveness for children’s ear infections and barriers to antibiotic-sparing approaches such as delayed antibiotics.

Healthcare utilisation is changing, with 19% of our sample consulting an online doctor and 29% a community pharmacy, and this survey was before the Pharmacy First scheme in England where pharmacists can supply medication for ear infections. Effective tools and training exist that can ensure consistency in the messaging for patients, but these need to be adapted and implemented across the care pathway. Examples that have reduced antibiotic prescribing include the TARGET (Treat Antibiotics Responsibly, Guidance, Education, and Tools) AMS (antimicrobial stewardship) training for primary care prescribers^38^ and an interactive booklet for parents on RTIs in children.^39^ Implementation of TARGET training is underway for general practices across England, which could support with uptake of existing resources. Additional resources to support parents with common infections exist, such as ‘Healthier Together’.^40^ Empowering parents with evidence-based information on managing their child’s ear infection may provide opportunities to reduce primary care consultations and consequently antibiotic use.

## Data

The dataset relied on in this article is available from the corresponding author on reasonable request.
